# Selective Hepatitis B Virus Vaccination Has Reduced Hepatitis B Virus Transmission in The Netherlands

**DOI:** 10.1371/journal.pone.0067866

**Published:** 2013-07-29

**Authors:** Susan Hahné, Robin van Houdt, Femke Koedijk, Marijn van Ballegooijen, Jeroen Cremer, Sylvia Bruisten, Roel Coutinho, Hein Boot

**Affiliations:** 1 Centre for Infectious Disease Control, National Institute for Public Health and the Environment, Bilthoven, The Netherlands; 2 Amsterdam Public Health Service, Amsterdam, The Netherlands; 3 Amsterdam Medical Center, Amsterdam, The Netherlands; 4 Utrecht University, Utrecht, The Netherlands; University of Cambridge, United Kingdom

## Abstract

**Background & Aims:**

In the Netherlands, a selective hepatitis B virus (HBV) vaccination programme started in 2002 for men having sex with men, drug users, commercial sex workers and heterosexuals with frequent partner changes. We assessed the programme's effectiveness to guide policy on HBV prevention.

**Methods:**

We analysed reports of acute HBV infection in the Netherlands between 2004 and 2010 requesting serum from patients for HBV-genome S- and C-region sequencing. We used coalescence analyses to assess genetic diversity of nonimported genotype-A cases over time.

**Results:**

1687 patients with acute HBV infection were reported between 2004 and 2010. The incidence of reported acute HBV infection decreased from 1.8 to 1.2 per 100,000 inhabitants, mostly due to a reduction in the number of cases in men who have sex with men. Men were overrepresented among cases with an unknown route of transmission, especially among genotype A2 cases mainly associated with transmission through male homosexual contact. The genetic diversity of nonimported genotype-A strains obtained from men who have sex with men decreased from 2006 onwards, suggesting HBV incidence in this group decreased.

**Conclusions:**

The selective HBV-vaccination programme for behavioural high-risk groups very likely reduced the incidence of HBV infection in the Netherlands mainly by preventing HBV infections in men who have sex with men. A considerable proportion of cases in men who did not report risk behaviour was probably acquired through homosexual contact. Our findings support continuation of the programme, and adopting similar approaches in other countries where HBV transmission is focused in high-risk adults.

## Introduction

Hepatitis B virus (HBV) is a major cause of liver disease and death primarily resulting from sequelae of chronic HBV infection including liver cancer and cirrhosis. The HBV is transmitted perinatally and through sexual and parenteral contact. An estimated 400 million people worldwide are infected with HBV, accounting for an estimated one million deaths annually [1], [2]. A safe and effective vaccine against HBV has been available since 1982 and is included in universal vaccination programmes in more than 170 countries [3].

The estimated prevalence of chronic HBV infection in the Dutch population of about 17 million inhabitants is 0.2%, which is among the lowest reported prevalences worldwide [4], [5]. Universal infant HBV vaccination was introduced in the Dutch national immunisation programme in August 2011 after an assessment by the Dutch Health Council that included a favourable cost-effectiveness analysis [6]. In addition to the recent introduction of universal vaccination, the Netherlands has a selective vaccination programme that provides HBV vaccination free of charge for population subgroups at increased risk of HBV due to risk behaviour, including men having sex with men (MSM), drug users, commercial sex workers and heterosexuals who change partners frequently. After a pilot programme in seven regions between 1998 and 2000, the permanent programme was implemented nationally from 2002 onwards. From 2007 onwards, heterosexuals with multiple partners were excluded from the target population as there was insufficient evidence of an increased risk in this group. From 2012 onwards, drug users were excluded for the same reason. Vaccination of drug users is now provided through drug treatment services.

Selective HBV vaccination of population subgroups at increased risk of HBV infection is recommended in all European countries and the USA [7], [8]. Irrespective of universal vaccination, programmes targeting behavioural high-risk groups will be needed for the coming decades, depending on the coverage achieved and the age groups targeted for universal vaccination. Selective programmes have, however, frequently been criticised as ineffective since the reported vaccine coverage achieved in the target populations was considered too low [9]–[12]. The impact on HBV transmission of selective vaccination programmes targeting behavioural high-risk groups has never been demonstrated on a national level.

Traditional epidemiological methods have several limitations in assessing the impact of selective vaccination. Vaccine coverage in the target populations is an obvious but problematic indicator, as estimates of the size of these hidden populations are very uncertain [13], [14]. Routine surveillance based on the reporting of acute HBV infection is incomplete, as less than half the cases of acute HBV infection among adults are symptomatic [15], not all patients with symptoms seek healthcare, and health care professionals do not manage to report all cases, reporting variably over time. Furthermore, for about a quarter of the cases, the route of acquisition of the virus remains unknown despite patient interviews [16]. Lastly, it is difficult to draw conclusions about the impact of vaccination since its effects can be counterbalanced by increased risk behaviour. Molecular epidemiological and phylodynamic methods take the genetic variability of a pathogen and its evolutionary processes into account, and as a result, low reporting quality and sampling intensity have less effect. These methods are therefore complementary to report-based surveillance in providing insight into the transmission dynamics of HBV [17].

A preliminary assessment of the selective vaccination programme in the Netherlands in 2007 showed that its effectiveness to reduce HBV transmission was limited [13]. Using a more complete dataset and applying additional analytical methods, we re-assessed the effectiveness of this programme to guide its implementation and to inform prevention and control of HBV in other countries where transmission is concentrated in risk groups.

## Patients and Methods

### Study population

We studied all reported cases of acute HBV infection in the Dutch population between 2004 and 2010. Cases of acute HBV infection are statutorily notifiable in the Netherlands. The case definition for reporting was a positive laboratory result for immunoglobulin M antibody to the hepatitis B core antigen (IgM anti-HBc) and/or hepatitis B surface antigen (HBsAg; the latter only after exclusion of hepatitis A and C viruses) in a person with an acute onset of symptoms compatible with acute hepatitis and jaundice and/or increased serum aminotransferase. Following reporting of cases by clinicians and laboratories, public health nurses interviewed patients with acute HBV infection to ascertain risk exposures and possible source(s) of infection. On the basis of the interview, the most probable mode of transmission, together with other patient data, was registered in an anonymous, on-line, national database. In the analyses, we distinguished the following transmission modes: heterosexual contact, male homosexual contact, injecting drug use, and ‘other’ modes of transmission (including parenteral procedures abroad, needle stick accidents, and multiple probable routes of transmission). If it was unknown whether the sexual contact was heterosexual or homosexual, the case was included in the unknown transmission route group. From 2004 onwards, we asked medical microbiology laboratories to submit serum samples from all patients with a reported case of acute HBV to one of the three participating laboratories [the National Institute for Public Health and the Environment (RIVM), the Public Health Laboratory Amsterdam, and the Erasmus Medical Centre in Rotterdam]. All laboratories isolated HBV DNA and a 648-nucleotide (nt) fragment of the S region. The RIVM only amplified and sequenced a 655 nt fragment of the C region as previously described [18], [19].

We described cases with the age and sex of the patient and the probable mode of transmission, and we studied trends over time. Differences in proportions were assessed for statistical significance using the chi-square test. We assessed trends in the number of case reports over time by Poisson regression with an identity link function, while trends in proportions over time were assessed by calculating a Chi-square for trend. Only significant trends (p<0.05) are reported. For significant trends we assessed whether there was a better fit to the data using a cubic spline. We assessed differences in continuous variables using the Wilcoxon rank sum test. Stata version 11.0 was used for these analyses (StataCorp, Texas).

Genotypes and genosubtypes were assigned to cases by entering their S-region sequences in a typing tool that uses reference strains as described by Norder et al. [20], [21]. We assessed clustering of strains by aligning sequences with BioEdit 7.0.9.0 [22], removing relatively short sequences. We used BioNumerics version 6.6 (Applied Maths, Sint-Martens-Latem, Belgium) to construct a maximum parsimony tree based on the S-region sequence. We selected a subgroup of cases for which both the S- and C-region sequences were available; these cases were of genotype A and were reportedly acquired in the Netherlands (i.e. nonimported). We distinguished two groups in this subset: MSM cases and cases acquired by heterosexual contact. We studied temporal changes of the effective population size of HBV causing acute infections in each of these groups by constructing Bayesian skyride plots in BEAST version 1.7.1 [23]. Hereby we assumed a general-time-reversible substitution model, a γ-shaped site heterogeneity model, and a relaxed molecular clock with a lognormal distribution. We studied the statistical support of any observed changes in the skyride plots over time by comparing its median posterior likelihood (quantified by its Bayes factor) with likelihoods from analyses assuming a constant population size. We considered differences greater than three in Bayes factors to be significant. All sequences used in our analysis were deposited in GenBank (accession numbers:

KC885087 - KC885557/KC885558 - KC885891)). Ethical approval was sought but deemed not necessary by the ethics committee of the Amsterdam Medical Centre. The reason for this was that residual sera collected for HBV diagnostic purposes were in this project used anonymously for HBV testing only. Informed consent by participants was therefore not part of the study procedures.

### Role of the funding source

The Netherlands Organisation for Health Research and Development (ZonMW) sponsored the study (research grant 125010004). This sponsor had no role in the study design, collection, analyses or interpretation of data, writing of the report or the decision to submit the paper for publication.

## Results

### Vaccination programme

Regional Public Health Services implement the selective vaccination programme for behavioural high-risk groups [24], which the Centre for Infectious Disease Control of the RIVM centrally coordinates. Vaccinations are given at diverse locations, including public health premises, in prisons, drug service locations, and outreach locations, including gay men's saunas and locations of commercial sex workers (Table 1). Serum is drawn from each participant for HBV serology (anti-HBc-IgG, and if positive, HBsAg) at the first vaccination visit. Anti-HBc-positive individuals do not receive subsequent vaccinations, while HBsAg-positive individuals are referred to routine clinical care. All vaccinations and serological test results are registered in an on-line, national database. From 1998 to 2011, about 105,000 individuals received at least one HBV vaccination within the programme. Of these, about one-third were MSM. On average, 19% of the MSM received their vaccinations in outreach locations. Nearly three-quarters of the HBV-susceptible MSM completed the series of three doses; 0·6% of the MSM were found to be chronically infected (Table 1).

**Table 1:** Characteristics of the selective HBV vaccination programme for behavioural high-risk groups, The Netherlands, 1998–2010.

	Men who have sex with men	Heterosexuals with frequent partner change†	Drug users	Commercial sex workers
**Number receiving first dose**	32,746	40,717	17,127	14,518
**Vaccination locations** ‡				
Public Health Service (%)	51·4	39·1	5·3	23·5
STD clinic (%)	27·4	29·8	2·5	10·7
Outreach§ (%)	19·0	9·0	10·2	59·9
Drug services (%)	0·3	0·7	57·5	0·5
Prison (%)	0·7	21·0	24·4	5·3
General Practice (%)	1·3	0·4	0·1	0·2
**Prevalence** ¶				
anti-HBc prevalence (%) [95% CI]	11·3 [11.0–11·7]	5.4 [5·1–5·6]	14·5 [13·9–15·0]	16·0 [15·4–16·7]
HBsAg prevalence (%) [95% CI]	0·6 [0·6–0·7]	0·6 [0.5–0·7]	0·8 [0·7–1·0]	1·2 [1·0–1·4]
**Compliance (%)** ∥	73·7	60·2	58·0	50·7

Anti-HBc = antibody to the hepatitis B core antigen; CI = Confidence interval; HBsAg = hepatitis B surface antigen; STD = sexually transmitted disease.

† Heterosexuals with frequent partner changes were no longer included in the programme from 2007 onwards.

‡ Proportion of the first vaccinations given at different locations, in percentages. Note: information about the vaccine location was missing for 3856 vaccinations.

§ Outreach locations included bars and saunas frequented by MSM, shelters for homeless people, and commercial sex worker locations.

¶ Prevalence was calculated by dividing the number of patients with a positive test by the total number with a test result.

∥ Compliance is defined as the proportion of those susceptible at the first vaccination completing three doses. Data up to 30 June 2011 were included.

### Reports of acute HBV infection

**Figure 1:**
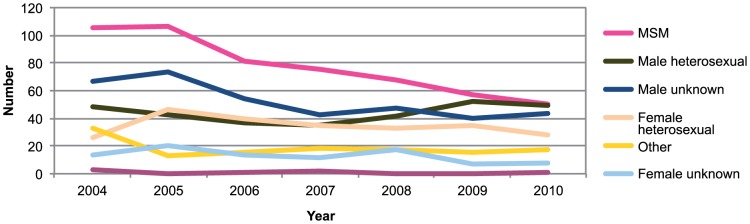
Number of cases of acute HBV infection by most probable mode of transmission and year reported in the Netherlands, 2004–2010 (N = 1687).

Between January 2004 and the end of December 2010, 1696 cases of acute HBV infection were reported in the Netherlands. We excluded nine cases because the gender of the patients was not reported, leaving 1687 patients, of whom 1320 (78·3%) were men. Male cases were older than female cases (median age 40 and 29 years, respectively; p<0·001). Six patients were reported to have died due to the HBV infection (0·4% of 1676 cases with information about survival), and 344 patients (21%) were admitted to hospital. The incidence of reports declined from 1.8 to 1.2 per 100,000 population between 2004 and 2010, mainly because of the declining incidence among men from 3.1 to 1.9 per 100,000. For women, the incidence remained constant at around 0.7 per 100,000. Of all patients, 78% were reported to have acquired the infection in the Netherlands. Male homosexual contact was the most frequently reported route of transmission (32% of the cases; Figure 1). Male patients with acute HBV infection more frequently reported an unknown route of transmission than female patients (20% and 5%, respectively, p<0.001).

Most of the decrease in the number of acute HBV reports could be attributed to a declining number of reports for MSM (Figure 1). The number of infected men with an unknown mode of transmission also declined from about 70 annually in 2004 and 2005 to about 45 annually from 2007 onwards. The number of cases in women with no route of transmission reported also declined (from 14 in 2004 to 8 in 2010). For none of these trends there was a better fit of a cubic spline compared to a linear trend (data not shown). There were only seven cases in injecting drug users (IDUs), three of which were reported in 2004. Relative to all cases reported by year, the proportion for MSM showed a decreasing trend between 2004 and 2010, while the proportion of male cases attributed to heterosexual contact increased (both p<0.05).

### Genotype analyses

S-region sequences were available for 902 of the 1687 reports (53%). For four additional cases, information about the genotype, but not the genosubtype, was available. The proportion of reports for which information about genotype was available varied by Public Health Service (ranging from 22% to 100%; p<0.001) and was lower for patients born abroad than for those born in the Netherlands (46% and 56%, respectively; p<0.01). The presence of genotype information did not vary by most probable mode of transmission, gender, or year of report (p>0.1).

**Figure 2:**
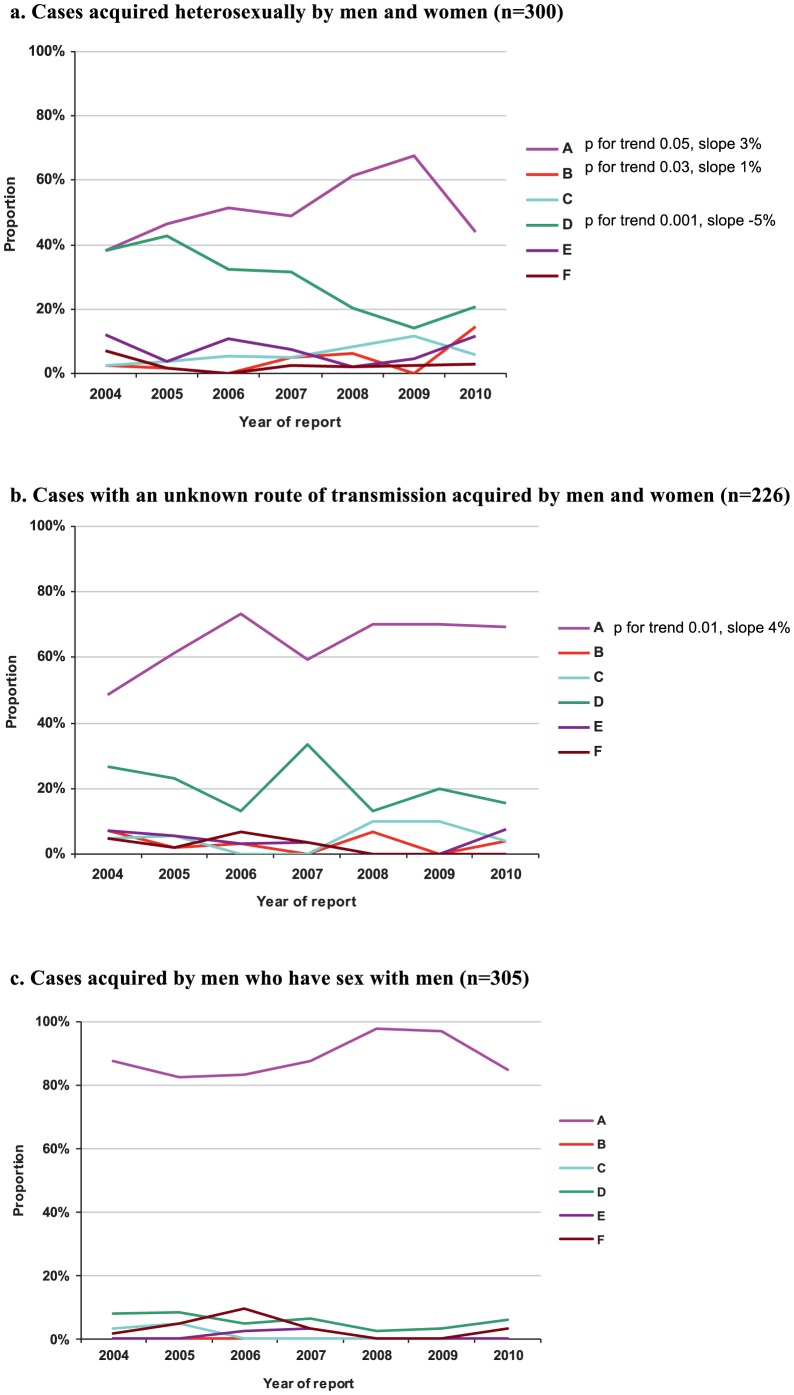
a–c. Genotype distribution by year of reporting of acute HBV infections, the Netherlands, 2004–2010. a. Cases acquired heterosexually by men and women (n = 300); b. Cases with an unknown route of transmission acquired by men and women (n = 226) c. Cases acquired by men who have sex with men (n = 305).

Genotype A was the most frequent genotype among the cases of MSM, for men and women with heterosexually acquired HBV, and cases with an unknown route of transmission (Figure 2a-c). Genotype D was the second most frequent genotype for all three of these subgroups, although it only accounted for a small proportion of cases among MSM. Among heterosexually acquired cases, the proportion of cases with genotype A and B increased over time until 2009, while the proportion that was genotype D decreased. Among cases with an unknown route of transmission, the proportion with genotype A increased . For none of these trends there was evidence for a better fit of a cubic spline compared to a linear function. The genotype distribution did not change among MSM. Men were over-represented among genotype A cases acquired by heterosexual or unknown routes of transmission (55% and 88%, respectively).

Genosubtype A2 was by far the most prevalent (94%) among genotype A cases. Its proportion varied by risk group, ranging from all cases in IDUs (2 cases) and 98% in MSM (261 cases) to 79% in women heterosexuals (54 cases; p<0.001). Genotype D1 was the most prevalent (66%) among genotype-D cases. This proportion did not differ by risk group (p = 0.4). The genotype-D3 strain that used to be associated with IDUs in the Netherlands was no longer detected [25].

**Figure 3:**
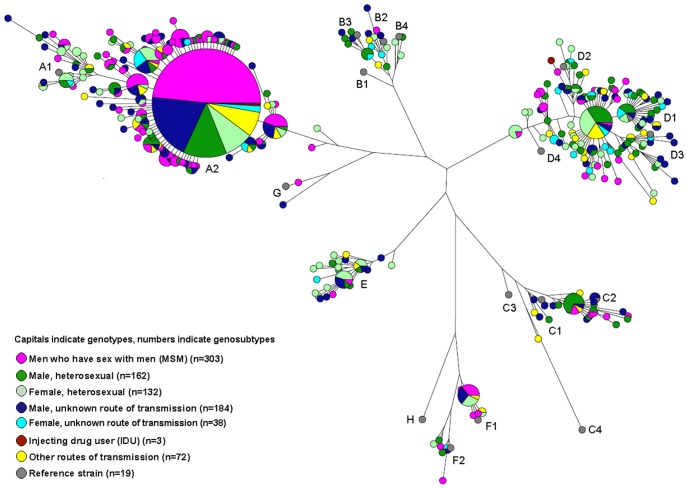
Maximum parsimony tree based on the HBV S-region sequence of acute cases of HBV infection (n = 894), by most probable mode of transmission and gender, in the Netherlands, 2004–2010, and selected reference strains (n = 19).

Phylogenetic analysis demonstrated a large cluster (353 cases) of indistinguishable strains within genotype A2 (Figure 3). Nearly half (48%) of the cases with this clonal strain concerned MSM; 13%, heterosexual men; and 9%, heterosexual women. Cases with this strain who did not report a route of transmission were 11 times more likely to be male than female (p<0.0001). The clonal strain was indistinguishable from a strain reported in the USA, the UK, and Japan [26]–[28]. For heterosexuals and those with an unknown route of transmission, a significantly increasing trend appeared in the proportion of cases carrying this clonal strain. There was no trend in the occurrence of this clonal strain among MSM.

### Coalescent analyses of genotype-A cases acquired in the Netherlands

**Figure 4:**
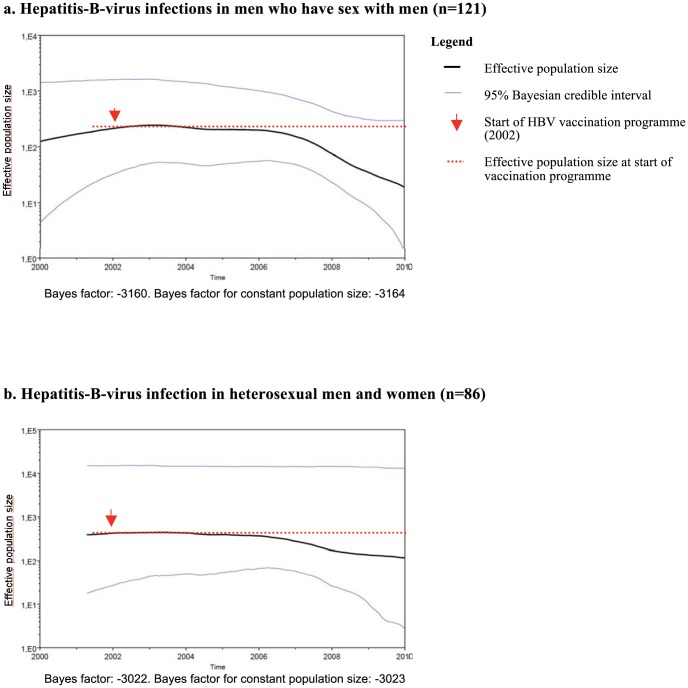
a,b. Bayesian skyride plot: estimated genetic diversity over time of the S- and C-region sequences of genotype-A acute hepatitis-B-virus infections acquired in the Netherlands, 2004–2010.

Both S-region and C-region sequences were available for 283 genotype-A cases acquired in the Netherlands. Of these, 121 were in MSM and 86 were heterosexually acquired HBV infections. All except three of the smaller Public Health Services reported the cases in this selection. The skyride plot of strains in cases of MSM showed a small increase in effective population size (i.e. genetic diversity) starting in 2000, followed by a large decrease from 2007 onwards. The plot for heterosexuals showed a small decrease starting from 2006 onwards (Figure 4a,b). Comparing the significance of the observed changes over time by comparing Bayes factors of the skyride analyses with those assuming a constant population size showed that only the changes in the plot for MSM were significant. This suggests HBV incidence fell among MSM; there is insufficient evidence for any change in risk among heterosexuals.

## Discussion

Our analyses of the largest molecular epidemiological study of acute HBV infection ever performed globally suggests that the Dutch selective vaccination programme for behavioural high-risk groups very likely reduced transmission of HBV in the Netherlands, primarily by reducing the HBV incidence among MSM. This is the first time effectiveness of selective HBV vaccination programme for adult high-risk groups has been demonstrated. The incidence of acute HBV reports diminished by about one-third in this period. Most of this decline was attributed to a reduction in the number of cases in MSM. This coincided with a marked decrease in the genetic diversity of HBVs sampled from MSM, suggesting that the incidence in this group had genuinely diminished. Taken together with the large number of MSM vaccinated against HBV since the start of the programme, we conclude that this programme has effectively reduced transmission of HBV among MSM. It does remain uncertain, however, which part of the observed decline in HBV transmission among MSM is due to direct or indirect (herd immunity) effects of the vaccination programme. It is possible that treatment of chronically infected MSM identified through the programme also contributed to preventing new infections.

Our results and conclusions contrast with an earlier evaluation of the Dutch selective HBV vaccination programme [13], probably because new methods were used to analyse more years of data. An evaluation of the programme in Amsterdam with data up to 2006 indicated that the programme was effective in preventing transmission among MSM, an effect that may not have been visible in case-based surveillance because it coincided with an increase in risk behaviour [17]. Our findings are important since selective vaccination programmes will need to continue for several decades, irrespective of universal HBV vaccination. Extending universal vaccination to adult populations as suggested in Japan is unlikely to be cost-effective [29].

The conclusion that the selective vaccination programme rather than possible reductions in risk behaviour reduced HBV transmission among MSM is corroborated by observations from surveillance data regarding other sexually transmitted infections and monitoring of risk behaviour among MSM. The annual number of reports of MSM with gonorrhoea doubled between 2004 and 2010 [30], [31]. Risk behaviour among MSM in the Amsterdam Cohort Studies steeply increased during the second half of the 1990s and early 2000s, then levelled off during our study period [32].

If we assume that all MSM in the Netherlands were in the target population for the selective vaccination programme [13] the estimated vaccination coverage would accrue to around 1% per year. Earlier modelling of HBV transmission among MSM in the Netherlands predicted that, with a coverage of 2% per year, the HBV incidence among MSM could be halved in 10 years if specifically MSM at high-risk of HBV acquisition were vaccinated [33]. Our observation that the number of reports of cases in MSM was halved after 9 years of programmatic HBV vaccination suggests that the programme was successful in reaching high-risk MSM. The large proportion of vaccines that were given at outreach locations may have been essential to this success. The relative absence of homophobia, marginalisation and stigmatisation of MSM in the Netherlands compared to most other countries in the world is likely to have had a beneficial effect on improving access to prevention services [34].

The distribution of genotypes sampled from MSM did not change over time, which confirms that HBV-vaccine-induced immunity protects against all genotypes [21]. In contrast, there was a relative increase of genotype A among heterosexuals up to 2009 and a decrease in genotype D. The clonal A2 strain that caused the main cluster of cases in MSM, and became increasingly frequent among heterosexuals and those with an unknown route of transmission. This strain has an S-region sequence that is identical to the clonal strain documented to have spread among adults in the UK, USA and Japan (the ‘UK prison variant’) [26]–[28]. This spread may be explained by factors including a relative advantage of this strain in causing chronic infection or a higher viral load [28], [35]–[37]. The decrease of genotype D among heterosexuals in our study population may have been caused by demographic changes in the Netherlands: migration from Mediterranean countries such as Morocco and Turkey, where genotype D is endemic [20], [21], has considerably decreased in the last decade [38]. The observation that the overall number of cases of HBV among heterosexuals did not increase after this group was excluded from the selective vaccination programme in 2007 provides a justification for this change in policy. There was a non-significant reduction of genetic diversity among heterosexually acquired cases starting after 2006. This indicates possible herd-immunity effects of the selective vaccination programme. It may also reflect the spreading of the clonal A2 strain in these populations.

Regarding the cases with an unknown route of transmission, the observation that men are over-represented, particularly among genotype A and subtype A2 strains, but even more so among the clonal A2 strain, suggests that a considerable proportion of these cases is acquired through male homosexual contact that is undisclosed in interviews with public health nurses. The decreasing trend over time in the number of men's cases with an unknown route of transmission that parallels the decrease in cases in MSM further corroborates this hypothesis.

In contrast with many European countries including the UK, Denmark, and the Baltic states [39]–[41], drug use no longer plays a role in the transmission of HBV in the Netherlands. Furthermore, the specific strain formerly associated with injecting drug use has disappeared. Explanations for this include the reduction of injection among drug users, ageing of the population, and high mortality among those at highest risk [25]. The effectiveness of the selective vaccination programme for preventing HBV among IDUs could not be assessed as the incidence of HBV among IDUs in the Netherlands had already decreased before the implementation of the programme in 2002.

The strength of our study is that it is based on nationwide, population-based, surveillance data of acute hepatitis B cases and hepatitis B viruses for a 7-year period. We maximised information obtained from the data by combining epidemiological analyses of cases and genotypes with phylodynamic analyses of viral sequences. The synergy of combining surveillance methods was used to evaluate the effectiveness of the selective vaccination programme and to generate hypotheses about viral characteristics that may favour transmission. Coalescence-based studies may be particularly important in situations where traditional epidemiological surveillance is less developed, e.g. in other countries or for other infections. Nevertheless, more methodological work is needed to further validate this method for different epidemiological situations.

Our study has some limitations. The proportion of cases for which a sequence was available varied considerably between Public Health Services. This was caused mainly by differences in local practices, e.g. how long serum samples taken for primary diagnostics were stored. The relatively few samples is not problematic for the coalescent analyses, as long as the sampling intensity is fairly constant over time [42]. Comparing the results of coalescent analyses with epidemiological analyses and observations from surveillance of behaviour and of another sexually transmitted disease proved the validity of these coalescent analyses. However, inferences from coalescence analyses have to be drawn with caution since they can lead to a biased estimate of the effective population size in certain conditions [43].

To conclude, we have demonstrated the synergy of combining report-based surveillance combined with molecular epidemiology and phylodynamics in understanding HBV transmission, evaluation of a public health programme, and generating hypotheses about viral characteristics. These methods are likely to be useful for many other infectious diseases and contexts. By doing so, we found evidence that the selective HBV vaccination programme for behavioural high-risk groups reduced HBV transmission in the Netherlands primarily by preventing infections among MSM. This supports continuation of the programme. It also provides important information to guide control of HBV infection in other countries where HBV transmission occurs predominantly in adult high-risk groups.
